# Prediction in cultured cortical neural networks

**DOI:** 10.1093/pnasnexus/pgad188

**Published:** 2023-06-27

**Authors:** Martina Lamberti, Shiven Tripathi, Michel J A M van Putten, Sarah Marzen, Joost le Feber

**Affiliations:** Department of Clinical Neurophysiology, University of Twente, PO Box 217 7500AE, Enschede, The Netherlands; Department of Electrical Engineering, Indian Institute of Technology, Kanpur 208016, India; Department of Clinical Neurophysiology, University of Twente, PO Box 217 7500AE, Enschede, The Netherlands; W. M. Keck Science Department, Pitzer, Scripps, and Claremont McKenna College, Claremont, CA 91711, USA; Department of Clinical Neurophysiology, University of Twente, PO Box 217 7500AE, Enschede, The Netherlands

**Keywords:** prediction, memory, mutual information, cortical neural networks

## Abstract

Theory suggest that networks of neurons may predict their input. Prediction may underlie most aspects of information processing and is believed to be involved in motor and cognitive control and decision-making. Retinal cells have been shown to be capable of predicting visual stimuli, and there is some evidence for prediction of input in the visual cortex and hippocampus. However, there is no proof that the ability to predict is a generic feature of neural networks. We investigated whether random in vitro neuronal networks can predict stimulation, and how prediction is related to short- and long-term memory. To answer these questions, we applied two different stimulation modalities. Focal electrical stimulation has been shown to induce long-term memory traces, whereas global optogenetic stimulation did not. We used mutual information to quantify how much activity recorded from these networks reduces the uncertainty of upcoming stimuli (prediction) or recent past stimuli (short-term memory). Cortical neural networks did predict future stimuli, with the majority of all predictive information provided by the immediate network response to the stimulus. Interestingly, prediction strongly depended on short-term memory of recent sensory inputs during focal as well as global stimulation. However, prediction required less short-term memory during focal stimulation. Furthermore, the dependency on short-term memory decreased during 20 h of focal stimulation, when long-term connectivity changes were induced. These changes are fundamental for long-term memory formation, suggesting that besides short-term memory the formation of long-term memory traces may play a role in efficient prediction.

**Significance Statement**

Prediction has been hypothesized to be one of the fundamental brain functions guiding our everyday actions. Here, prediction means that present neural activity reduces the uncertainty of upcoming external sensory inputs. Theoretical work and studies of specific neural networks have provided some first evidence of prediction but is this a generic network feature? Can random neuronal networks predict, and if so, how is prediction related to memory? We used mutual information (MI) to show that in vitro networks of dissociated neurons can predict upcoming external stimuli, and that prediction depends on short-term memory. This dependency decreases with time when long-term connectivity changes are induced, suggesting that long-term memory is also involved in efficient prediction.

## Introduction

Our ability to perform action and make decisions has long been the topic of extensive research. It has been suggested that memorization and prediction are two of the most important functions guiding our actions (1, 2). Prediction can be defined as the ability to reduce uncertainty in afferent input signals from the external world and has been shown to critically depend on memory (3). In particular, prediction requires memory, although one can memorize without predicting (4). It has been hypothesized that our memory is in service of prediction (5), so that we only memorize certain features of the stimulus. Memory can be divided into short-term memory (time scales of seconds) and long-term memory (time scales of minutes or longer). Short-term memory can be very useful for prediction. In particular, the registration of recent external inputs plays a fundamental role in prediction (6–9, 3). It has been theorized that the successful conclusion of an action is mainly related to the network ability of making predictions (1, 2). In essence, neural networks seem to make continuous sensory predictions which guide their actions (1, 2, 5, 10–15) and which, if operating efficiently, require storage of just the right memories.

Technological advances and the use of in vitro neural networks have contributed to progress in understanding the neuronal mechanisms underlying memory and prediction (16, 17). The use of multielectrode arrays (MEAs) enables recording of network activity from in vitro neural cultures, and facilitates deeper investigation of these functions at a network level (16, 18). Activity and connectivity in these networks mutually affect each other, and the observation that both stabilize in mature cultures suggests that networks develop an equilibrium between activity and connectivity (19, 20). Repeated electrical stimulation of a specific subset of neurons (focal stimulation) has been shown to induce new activity patterns that exert a driving force away from the existing equilibrium. This results in long-term connectivity changes (20), which are interpreted as the formation of long-term memory traces (21, 22). Random electrical stimulation or global optogenetic stimulation did not induce significant connectivity changes, possibly because it did not induce specific new activity patterns necessary to drive networks away from the existing equilibrium (21, 23).

MI estimates how much one signal reduces the uncertainty in another one (24) and provides a tool to quantify prediction (5). MI between recorded neuronal activity and past external stimuli provides a quantification of short-term memory, whereas MI between activity and future stimuli quantifies prediction (5). MI is based on the knowledge of the Shannon entropy of the considered signals (24, 25). This means that we first need to know the information contained in the sequence of given stimuli and in the recorded neuronal activity (5, 25). According to Fano’s inequality, this MI tells how much the probability of error in predicting or memorizing the input signal decreases when we know the neural response.

Most studies aiming to elucidate the role of prediction in network functioning have been theoretical (1–3), although some promising experimental steps have been made on retinal ganglion cells (5, 26–29), which were shown to predict movements of objects (5, 26–28). In addition, there are other indicators that there is prediction in the brain, from the spatiotemporal receptive fields in the primary visual cortex (10) to the response timescales of neurons in the hippocampus (30, 11) to hierarchical properties of neural coding (31). If prediction is indeed a guiding principle in our actions, networks of neurons in general should be able to predict. However, proof that random neuronal networks without evolved wiring predict is still missing.

To that end, we investigated the hypothesis that random networks of in vitro rat cortical neurons can predict, and that prediction depends on short-term memory. In addition, we examined possible involvement of long-term memory. To answer these questions, we stimulated cultured neural networks either focally (electrically) or globally (optogenetically) to induce long-term memory traces or not. We used MI to determine to what extent network activity predicts upcoming stimuli and how it uses memory to do so. Then, we determined the association between MI estimates of prediction and of short-term memory, and how this changes with the induction of long-term connectivity changes. Finally, we determined how well in vitro neural networks predict, compared to a derived theoretical optimum.

## Materials and methods

### Culture preparation

Cortical neurons were obtained from Newborn rats and dissociated by trypsin treatment and trituration. About 50,000 cells (60 μl suspension) were plated on MEAs (Multi Channel Systems, Reutlingen, Germany), precoated with poly ethylene imine. MEAs contained 60 titanium nitride electrodes (diameter: 30 μm; pitch: 200 μm) and had a circular chamber (diameter: 20 mm) glued on top to create a culture well. The well was filled with ≈1 ml of R12 medium (32). MEAs were stored in an incubator, under standard conditions of 36${}^{\circ }$C, high humidity, and 5% CO${}_{2}$ in air. Culture medium was refreshed twice a week by removing 500 μl of the old medium and adding 550 μl of fresh medium, thus compensating for evaporation. To allow network maturation, all cultures were grown for at least 3 weeks before experiments started (33, 34, 19). For experiments, each culture chamber was firmly sealed with watertight but O${}_{2}$ and CO${}_{2}$ permeable foil (MCS; ALA Scientific). Then, MEAs were placed in a measurement setup outside the incubator. In this setup, high humidity and 5% CO${}_{2}$ were maintained. Recordings began after an accommodation period of at least 15 min. After experimenting, cultures were returned to the incubator.

To enable global optogenetic stimulation, cells were transfected with an adeno-associated virus (serotype 2.1), obtained from Penn Vector Core, Philadelphia, PA, USA. This viral vector contained the ChannelRhodopsin-2 gene, driven by the CaMKIIα promoter, which is found exclusively in excitatory neurons. The ChannelRhodopsin-2 gene contains a mutation (H134R) which makes it sensitive to blue light 470 nm (35). The initial volume of the virus with a physical titer of ≈1.31⋅1013 GC/ml was diluted 100 times in DPBS, and cultures were transduced with 25 or 50 μl the day after plating. Effective transduction was verified before recording by the appearance of the red fluorescent protein mCherry throughout the culture, and clear responses to 100 ms blue light pulses during experimental recording. This ChR2 variant shows a stable response to stimuli, facilitating global activation of networks throughout 20 h experiments, and sufficient temporal precision (≈15 ms) (36–38), given that interstimulus intervals were relatively high (>1 s), and network activity was analyzed in 100 ms time bins. All surgical and experimental procedures were approved by the Dutch committee on animal use (Centrale Commissie Dierproeven; AVD110002016802) and complied with Dutch and European laws and guidelines. Results are presented in compliance with the ARRIVE guidelines.

### Recording set-up

To record activity, we placed each MEA in a setup outside the incubator, consisting of a MC1060BC preamplifier and FA60s filter amplifier (both MultiChannelSystems GmbH, Reutlingen, Germany). Network signals are recorded from 59 electrodes using a custom-made Lab-View program, with a sampling frequency of 16 kHz per electrode. All analog signals were band-pass filtered (second-order Butterworth 0.1–6 kHz) before sampling (23). A detection threshold was set at 5.5 times the estimated root mean square noise level (ranging ≈3–5 μV). The noise estimation was continuously updated during recordings for each electrode. Whenever signals exceeded the detection threshold, a time stamp, the electrode number, and the wave shape (6 ms) were stored. A wave shape based algorithm was adapted from (39) for off-line artifact detection and removal. Electrodes typically recorded activity from one or more neurons, but we did not apply spike sorting. The reliability of this waves shape based method is doubtful, as the shapes of action potentials from individual neurons can substantially change, e.g. during intense firing in network bursts (40, 41). Thus, we analyzed activity recorded from small groups of neurons, rather than from individual neurons (22, 23).

### Focal and global stimulation

Either focal or global stimulation was applied during a stimulation period of 20 h, which was preceded and followed by 1 h of spontaneous activity recording. Focal stimulation was applied trough electrical stimulation to one electrode, using biphasic rectangular current pulses of 200 μs per phase (22). After probing all electrodes at various amplitudes (16–24 μA), one electrode was selected for stimulation, using the lowest amplitude that induced a network response after at least 50% of all stimuli. Amplitudes were low enough to avoid electrolysis. Stimuli were separated by interstimulus intervals (ISI), which were read from a pregenerated list, with (ISIs) drawn independently and identically from a density distribution designed to produce long-ranged temporal correlations. The resulting stochastic process was generated by a hidden Markov model (see Supplementary Material) and is an infinite-order Markov process (42). Networks need some time to recover from a stimulus response, and stimulus responses have been shown to decrease with high stimulation frequencies (39). We therefore set the average stimulation frequency of the ISIs to 0.2 Hz, with a minimum of 1 s between consecutive stimuli. For global (optogenetic) stimulation, power LEDs on a SinkPAD-II 20 mm Star Base (Blue (470 nm) 74 lm@700 mA from Luxeon-StarLEDs) were placed approximately 7 cm above the top of the MEA, with a Faraday cage, based on a stainless steel mesh, placed between the LED and the MEA to reduce electrically induced artifacts by the LED power cables (35). The duration and intensity of light pulses were set to induce a network response with a reliability of at least 50% (typically light pulses had 100 ms pulse width and ≈2.5 klx intensity, which translates to ≈3.5 mW/cm${}^{2}$). Interstimulus intervals were read from the same list as the one used for focal electrical stimulation.

### Mutual information

MI quantifies how much one signal reduces the uncertainty of another one. We calculated MI between neuronal activity *X* and the stimulation signal *S*. Recorded activity was first divided into bins of 100 ms, and then transformed into a series of N binary words (one word for each bin)


(1) X=x(1),x(2),…,x(N).

In each binary word, $x_{i}(n)$ was set to 1 if neuron i was active in the *n*th time bin, otherwise, $x_{i}(n)$ was set to 0. Thus, given we had recordings from 59 electrodes, activity could be encoded by a maximum of $2^{59}$ different words. The same binarization was applied to the stimulus signal (*S*)


(2) S=s(1),s(2),…,s(N).

For each time bin, we set s(n)=1 if there was a stimulus and s(n)=0 otherwise. After binarization, the entropies of the neuronal activity, H(X), and of the stimulation vector H(S), were calculated using the centered Dirichlet mixture estimator (25). This is a Bayesian estimator using a prior created specifically for binary data to deal with potential undersampling issues. Here, we used the Dirichlet–Synchrony estimator (25). Once both H(X) and H(S) were estimated, MI was calculated as


(3) MI(S;X)=H(S)+H(X)−H(S,X),

where H(S,X) is the joint entropy of *X* and *S*. To reduce the computational load of calculating MI between recorded activity *X* and the stimulation vector *S* and to reduce errors associated with undersampling, we greedily approximated the MI between neural activity and stimulus by the MI between the neural activity of the best five neurons and the stimulus. We first calculated MI between activity from single electrodes $X_{i}$ and *S*. Next, we combined the electrode with maximum MI with all others to find the best combination of two. This procedure was repeated to obtain a set of maximum five electrodes with the highest MI between their activity *X* and *S*. The set size was limited to five electrodes because the extra information provided by each additional electrode rapidly decreased, and more electrodes would unnecessarily increase computational load. This procedure may lead to underestimation of MI, which was regarded as a lower limit.

### Data analysis

**Fig. 1.**
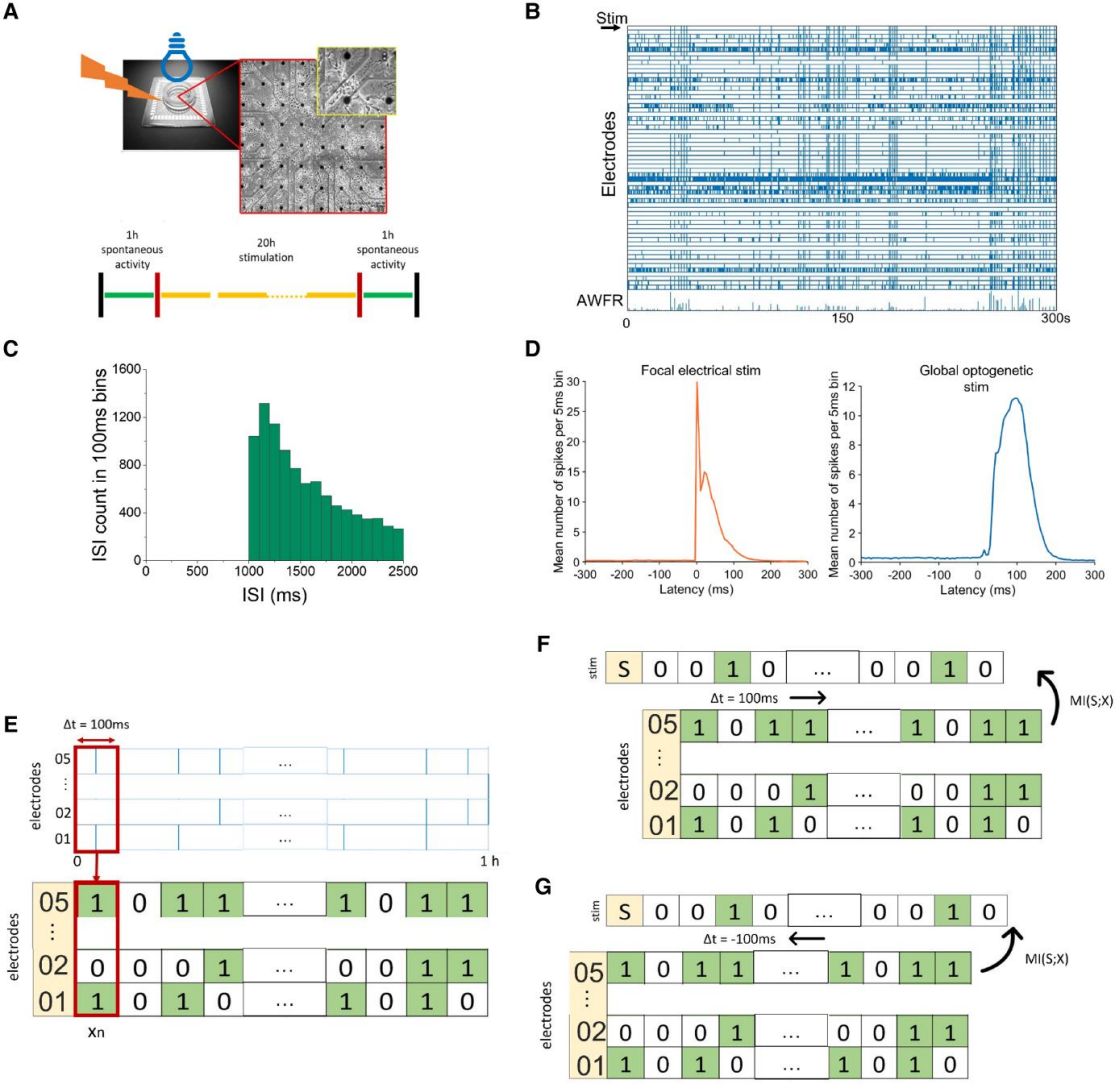
Example of recorded activity, stimulus responses, and application of MI. A) Example of MEA with cultured neuronal networks and stimulation protocol. B) Raster plot of recorded activity. each vertical tick indicates an action potential recorded at the corresponding electrode. Stimulation is indicated in the top row. C) Distribution of interstimulus intervals. D) Examples of average stimulus responses as obtained during 1 h of focal electrical (left) or global optogenetic stimulation (right). E) illustration of recorded activity (top panel, blue vertical lines represent action potentials) transformed into binary vectors (x(n)). Time is divided into bins of 100 ms. $x_{i}(n)$ is set to 1 if electrode i was active in that time bin, otherwise it is set to 0 (bottom panel). F, G) Mutual information is calculated between time shifted activity $X_{t+\Delta t}$ and unshifted stimulation $S_{t}$. Binarized activity is shifted forward (Δt>0) to quantify prediction (F) and backward (Δt<0) to short-term memory (G).

All cultures were tested for activity and stimulus responses before experimenting. Active electrodes were defined as electrodes that recorded at least 250 spikes in the first hour of spontaneous activity. Only cultures with at least ten active electrodes and clear responses to stimulation (example in Fig. 1) were used. A response was considered clear if the post-stimulus time histogram (PSTH) values, after the stimulus was sent, were higher than the averaged area before the stimulation plus 5 times its standard deviation. We used 20 cultures, which were stimulated electrically (n=10) or optogenetically (n=10). In total three cultures were excluded for lack of responsiveness to stimulation during the last 3 h (two electrically stimulated, one optogenetically). Details on performed experiments can be found in Supplementary material (Table S1). Part of these data has been used in a previous study for other goals (23).

#### Quantification of prediction and short-term memory

After verifying the effectiveness of stimulation throughout experiments (see Supplementary material), for every hour we analyzed the recorded data and calculated MI between *S* and various time shifted versions of *X*. To quantify prediction *X* was shifted Δt ms (Δt = n of bins ×100 ms) forwards before computing ${\mathrm{MI}}_{\mathrm{future}}$


(4) $${\mathrm{MI}}_{\mathrm{future}}(S;X)=\mathrm{MI}(S_{t};X_{t+\Delta t})\;\forall \;100\leq \Delta t\leq 2,000\;\mathrm{ms},$$

where $S_{t}$ and $X_{t+\Delta t}$ represent the unshifted stimulation signal and the time shifted binarized activity. The maximum time shift of 20 bins corresponds to 2 s. To quantify short-term memory, *X* was shifted Δt ms backwards before calculating ${\mathrm{MI}}_{\mathrm{past}}$, as illustrated in Fig. 1E–G,


(5) $${\mathrm{MI}}_{\mathrm{past}}(S;X)=\mathrm{MI}(S_{t};X_{t+\Delta t})\;\forall \;-1000\leq \Delta t\leq 0\;\mathrm{ms}.$$

Equations 4 and 5 yield ${\mathrm{MI}}_{\mathrm{future}}$ and ${\mathrm{MI}}_{\mathrm{past}}$ as functions of Δt.

#### Contribution of stimulus responses to prediction

To determine the contribution of stimulus responses to prediction, we masked the stimulus response in the neuronal activity. We substituted all binary words in the bin of stimulation and the three following bins by binary words randomly taken from periods with no stimulation of the same experiment to construct $X_{\mathrm{masked}}$. We calculated MI between *S* and $X_{\mathrm{masked}}$ for positive and negative time shifts to compute ${\mathrm{MI}}_{\mathrm{masked}}^{\;f}$ and ${\mathrm{MI}}_{\mathrm{masked}}^{\;p}$, respectively:


(6) $${\mathrm{MI}}_{\mathrm{masked}}^{f}={\mathrm{MI}}_{\mathrm{future}}(S,X_{\mathrm{masked}})$$

(7) $${\mathrm{MI}}_{\mathrm{masked}}^{p}={\mathrm{MI}}_{\mathrm{past}}(S,X_{\mathrm{masked}}).$$

In addition, for each hour, we determined the intrinsic predictive information of the stimulus itself (${\mathrm{MI}}_{\mathrm{self}}$) by calculating MI between the time shifted stimulation vector and the unshifted vector. (See Supplementary material for the derivation of ${\mathrm{MI}}_{\mathrm{self}}$.) If prediction would completely depend on the presence of clearly recognizable stimulus responses, with 100% stimulation efficacy, and no spontaneously occurring patters equal to the stimulus response, ${\mathrm{MI}}_{\mathrm{future}}$ should equal ${\mathrm{MI}}_{\mathrm{self}}$. We subtracted ${\mathrm{MI}}_{\mathrm{self}}$ from ${\mathrm{MI}}_{\mathrm{future}}$ to see to what extent ${\mathrm{MI}}_{\mathrm{future}}$ differed from ${\mathrm{MI}}_{\mathrm{self}}$. We used a computational model (details in Supplementary material) to investigate whether prediction requires synaptic transmission.

#### Relationship between prediction and memory

To assess the relationship between prediction and short-term memory, we used unmasked data to calculate the area under MI curves for positive (100–2,000 ms; $\Sigma {\mathrm{MI}}_{\mathrm{future}}$) and negative Δt (−1,000−−0 ms; $\Sigma {\mathrm{MI}}_{\mathrm{past}}$):


(8) $$\Sigma {\mathrm{MI}}_{\mathrm{future}}=\sum_{\Delta t=100}^{2,000\;\mathrm{ms}}{\mathrm{MI}}_{\mathrm{future}}(S,X)$$

(9) $$\Sigma {\mathrm{MI}}_{\mathrm{past}}=\sum_{\Delta t=-1,000}^{0\;\mathrm{ms}}{\mathrm{MI}}_{\mathrm{past}}(S,X).$$

Both parameters were calculated per each of the 20 h of stimulation and per single experiment. Then, we checked per each stimulation modality the relationship between $\Sigma {\mathrm{MI}}_{\mathrm{future}}$ and $\Sigma {\mathrm{MI}}_{\mathrm{past}}$. We fitted a linear equation and determined the correlation coefficient between the two. Finally, we investigated whether and how slope and offset of the fitted linear equations changed during the 20 h of stimulation. In addition, we also investigated the induction of possible connectivity changes by either electrical (focal) or optogenetic (global) stimulation following (23). Details on this analysis can be found in Supplementary material.

#### How efficiently do cultures predict?

To assess how well neural networks predict, we used the concept of the predictive information bottleneck, in which we determine the minimum information about the past ($I_{\mathrm{mem}}$) that is needed to maximize predictions about the future ($I_{\mathrm{pred}}$). Instead of working with the entire past and entire future, we replace the entire past of the stimulus at each time point by the time since last stimulus, $S^{+}$, and the entire future by the time to next stimulus, $S^{-}$ (examples on $S^{+}$,$S^{-}$, $I_{\mathrm{mem}}$ and $I_{\mathrm{pred}}$ can be seen in Fig. S5, Supplementary Material) (43):


(10) $$I_{\mathrm{mem}}=I[S^{+};X],\;I_{\mathrm{pred}}=I[S^{-};X].$$

We calculated minimum memory required to achieve certain predictive power. This yields the border between achievable and unachievable combinations of memory $I_{\mathrm{mem}}$ and predictive power $I_{\mathrm{pred}}$ as:


(11) $$R(D)=\min_{\;p(x|s^{+})\;:\;I[X;S^{-}]\geq D}I[X;S^{+}].$$

Here, *D* is the required predictive power, R(D) is the memory required to achieve that predictive power, *x* is a realization of *X*, $s^{+}$ is a realization of $S^{+}$, and $p(x|s^{+})$ is the conditional probability of neural activity given time since last stimulus. For further details, see Supplementary material.

### Statistical analysis

Normality of distributions was assessed by Shapiro–Wilk tests. In case of normality, group means ± standard error of the mean are presented. Significance of temporal differences were analyzed by one-way ANOVA (if normally distributed) or Kruskal–Wallis (if non-normal). Significance of correlation was analyzed using *t*-statistics. *P*-values <0.01 were considered to indicate significance. *T*-statistics measure was also applied to check trends of data. All statistical analysis were performed using SPSS statistics for Windows (IBM, Inc., Chicago, IL, USA) or Matlab (The Mathworks, Inc., Natick, MA, USA).

## Results

We first show that the activity recorded from cultured cortical networks provides information on past, as well as future stimulation. Then, we will show that most of the information on future stimuli is encoded in stimulus responses, which outlast the stimuli by several hundreds of milliseconds, and can be seen as a form of short-term memory. The dependency of prediction on short-term memory decreases during the induction of long-term connectivity changes by focal electrical stimulation, but not during global optogenetic stimulation, which did not induce connectivity changes. In all Figures orange refers to focal electrical stimulation, and blue to global optogenetic stimulation.

### Quantification of prediction and short-term memory

We used activity recorded from eight cultures that were focally stimulated and nine cultures (for details on amount of experiments see Supplementary material) that were globally stimulated during 20 h to calculate ${\mathrm{MI}}_{\mathrm{future}}$ and ${\mathrm{MI}}_{\mathrm{past}}$ as function of Δt (Eqs. 4 and 5). These cultures were plated from 12 independent cell suspensions obtained from different rats.

#### Focal stimulation

**Fig. 2.**
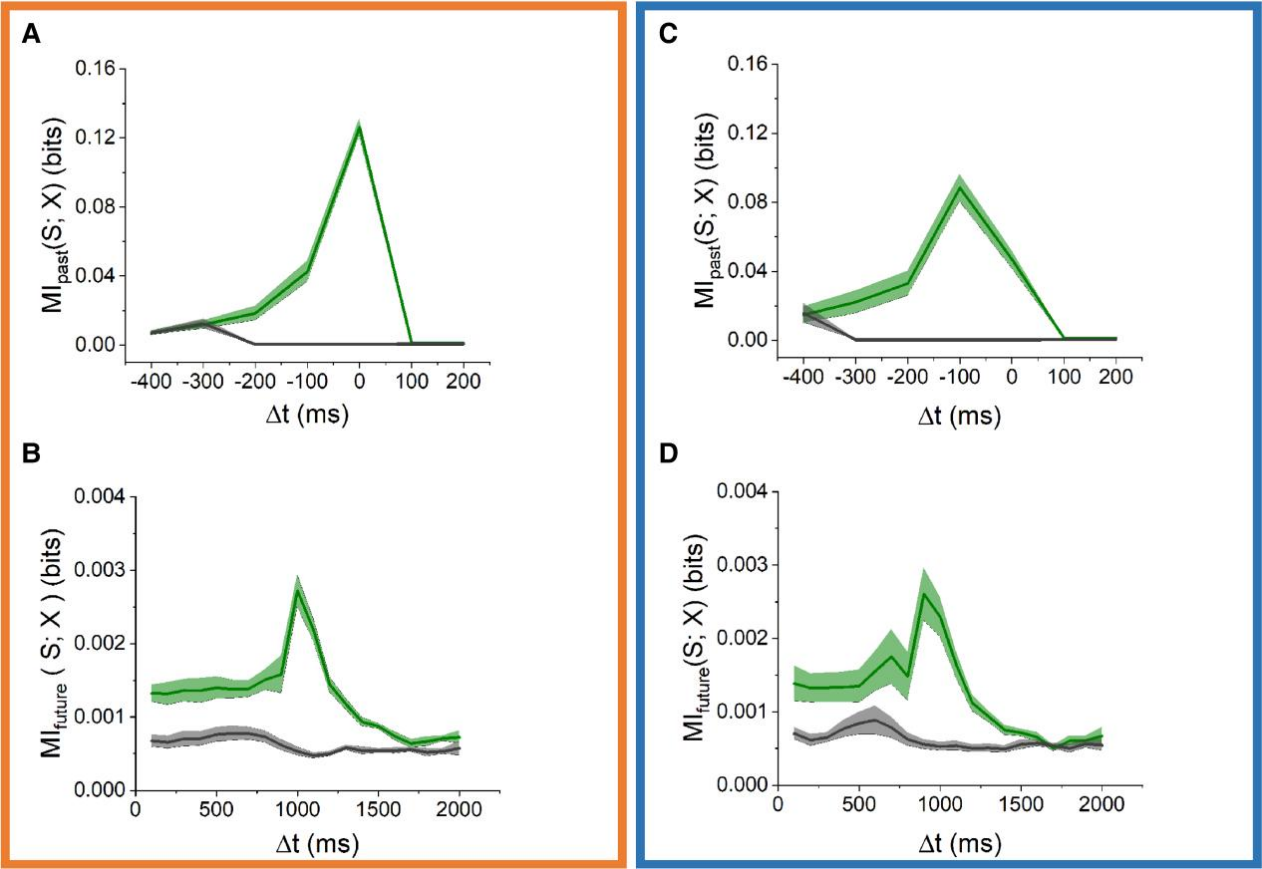
MI between activity and focal electrical stimulation (left panels) or global optogenetic stimulation (right panels). Upper panels A, C) show estimated short-term memory (${\mathrm{MI}}_{\mathrm{past}}$, green). The peaks in the interval Δt=[−100−−0] ms (A) or Δt=[−200−−100] ms (C) correspond to the immediate stimulus-response, and disappear after masking (${\mathrm{MI}}_{\mathrm{masked}}^{p}$, gray). Bottom panels B, D) show estimated prediction (${\mathrm{MI}}_{\mathrm{future}}$, green), with clear peaks around Δt=1100 ms (B) or Δt=1,000 ms (D). Both disappear after masking (${\mathrm{MI}}_{\mathrm{masked}}^{f}$, gray). Shaded areas indicate SEM and represents differences between experiments.

Fig. 2 shows ${\mathrm{MI}}_{\mathrm{future}}$ and ${\mathrm{MI}}_{\mathrm{past}}$, which quantify prediction and short-term memory, respectively. ${\mathrm{MI}}_{\mathrm{past}}$ shows a clear peak that closely corresponds to the time course of induced stimulus responses in 0–3 bins (meaning up until 300 ms) after each stimulus (Fig. 2A). ${\mathrm{MI}}_{\mathrm{future}}$ shows a plateau until Δt=1000 ms, followed by a clear peak at Δt=1,100 ms, corresponding to the most probable inter stimulus interval (see ISI distribution Fig. 1C). The peak is then followed by a decrease for 1,100<Δt<2,000 ms, reflecting the probability distribution of ISIs . ${\mathrm{MI}}_{\mathrm{future}}$ and ${\mathrm{MI}}_{\mathrm{past}}$ as functions of Δt hardly changed during 20 h of stimulation (see Supplementary material, Fig. S2).

#### Global stimulation

Fig. 2 shows that global stimulation resulted in ${\mathrm{MI}}_{\mathrm{future}}$ and ${\mathrm{MI}}_{\mathrm{past}}$ curves similar to the focal electrical stimulation results. With optogenetic (global) stimulation, ${\mathrm{MI}}_{\mathrm{past}}$ curves peaked at Δt=−100 ms, due to the longer latency of optogenetically induced stimulus responses. ${\mathrm{MI}}_{\mathrm{future}}$ showed a plateau for 100≤Δt≤900 ms, followed by a peak at Δt=1000 ms and a subsequent decrease (Fig. 2D). Both curves hardly changed during 20 h of stimulation.

### Contribution of stimulus responses to prediction

Stimulus responses were masked in the binarized activity X. Fig. 2A and C shows that ${\mathrm{MI}}_{\mathrm{masked}}^{p}$ was very close to zero for all −100≤Δt≤−400 ms, which confirms adequate masking of stimulus responses. To determine the contribution of stimulus responses to prediction, we used Eq. 6 to calculate ${\mathrm{MI}}_{\mathrm{masked}}^{f}$. In addition, we calculated the intrinsic predictive information of the stimulus itself (${\mathrm{MI}}_{\mathrm{self}}$; see Supplementary material) to determine whether and how that deviated from ${\mathrm{MI}}_{\mathrm{future}}$.

#### Focal stimulation

The peak at Δt=1100 ms as present in ${\mathrm{MI}}_{\mathrm{future}}$ was not visible in ${\mathrm{MI}}_{\mathrm{masked}}^{f}$ (Fig. 2B, dark gray). Very similar results were find for all analyzed hours. In addition, we calculated ${\mathrm{MI}}_{\mathrm{self}}$ curves (see Supplementary material) for every hour, which were subtracted from ${\mathrm{MI}}_{\mathrm{future}}$. This also eliminated the peak at Δt=1,100 ms in all hours.

#### Global stimulation

Fig. 2D shows, in dark gray, ${\mathrm{MI}}_{\mathrm{masked}}^{f}$ (Eq. 8) for globally stimulated cultures. Again, the ${\mathrm{MI}}_{\mathrm{masked}}^{p}$ and ${\mathrm{MI}}_{\mathrm{masked}}^{f}$ peaks disappeared, with no clear differences between analyzed hours. Subtraction of ${\mathrm{MI}}_{\mathrm{self}}$ from ${\mathrm{MI}}_{\mathrm{future}}$ again resulted in the disappearance of the peak at Δt=1000 ms, with no clear difference between the hours (see Supplementary material).

### Relationship between prediction and memory

We quantified prediction by $\Sigma {\mathrm{MI}}_{\mathrm{future}}$, (Eq. 8) and short-term memory by $\Sigma {\mathrm{MI}}_{\mathrm{past}}$, (Eq. 9), and investigated their relationship for focal as well as global stimulation.

#### Focal stimulation

**Fig. 3.**
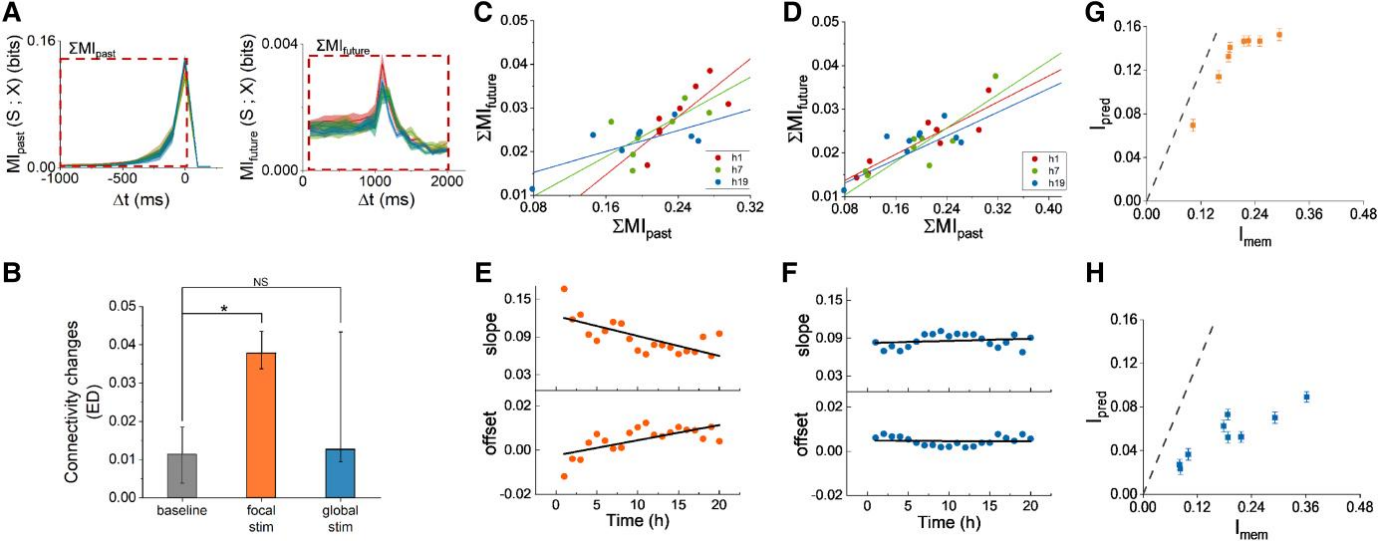
Relation between prediction and memory. A) Example of ${\mathrm{MI}}_{\mathrm{past}}$, ${\mathrm{MI}}_{\mathrm{future}}$ and the summation intervals to compute $\Sigma {\mathrm{MI}}_{\mathrm{past}}$ and $\Sigma {\mathrm{MI}}_{\mathrm{future}}$. B) Long-term connectivity changes induced by focal electrical stimulation (orange, p<0.007), and by global optogenetic stimulation (blue, p>0.2). Here, data were not normally distributed thus we show the median and the error bars represents the 75 and 25 percentiles. C) Typical examples of the relationship between $\Sigma {\mathrm{MI}}_{\mathrm{future}}$ and $\Sigma {\mathrm{MI}}_{\mathrm{past}}$ obtained with focal stimulation. C, D) Different colors represent different hours (inset). D) Examples of the relationship between $\Sigma {\mathrm{MI}}_{\mathrm{future}}$ and $\Sigma {\mathrm{MI}}_{\mathrm{past}}$ obtained with global stimulation. E, F) Trends of correlation coefficient R, slope and offset of the relation between $\Sigma {\mathrm{MI}}_{\mathrm{future}}$ and $\Sigma {\mathrm{MI}}_{\mathrm{past}}$, for focal (E) or global stimulation (F). When focally stimulated, slope decreased significantly with time (p<0.001), and offset increased significantly (p<0.001). In contrast, for global optogenetic stimulation slope and offset did not change in time (p>0.4). Black lines show the trend of slope and offset. In all panels $\Sigma {\mathrm{MI}}_{\mathrm{past}}$ and $\Sigma {\mathrm{MI}}_{\mathrm{future}}$ refer to Eqs. 9 and 8. Panels G,H) refers to the efficiency of predictions. In both panels the dashed line represents the theoretical optimum R(D) (see Supplementary material for the derivation). Focally stimulated cultures (G) reached 66.7±8% of their theoretical optimum, globally stimulated cultures (H) reached 30.4±6%.

When focally stimulated, $\Sigma {\mathrm{MI}}_{\mathrm{future}}$ was linearly associated with $\Sigma {\mathrm{MI}}_{\mathrm{past}}$ with an average correlation coefficient of 0.8±0.08 (range: 0.6–0.99), and no clear changes during the 20 h of stimulation. When fitted by a linear equation, the slope significantly decreased (p<0.001) with time, while the offset increased (p<0.001, see Fig. 3E), indicating a decreasing dependency of $\Sigma {\mathrm{MI}}_{\mathrm{future}}$ on $\Sigma {\mathrm{MI}}_{\mathrm{past}}$. In addition, electrical stimulation induce significant connectivity changes in the network (p<0.007, Fig. 3B).

#### Global stimulation

When globally stimulated, $\Sigma {\mathrm{MI}}_{\mathrm{future}}$ was highly correlated with $\Sigma {\mathrm{MI}}_{\mathrm{past}}$, with average correlation coefficient 0.9±0.03 (range: 0.8–1), and no clear differences between hours. Linear equations were fitted for each hour. Fig. 3F shows that slope (p>0.4) and offset (p>0.8) did not significantly change during the 20 h of stimulation. In addition, global optogenetic stimulation did not induce significant connectivity changes in the network (p>0.2, Fig. 3B).

### How efficiently do cultures predict?

To determine how well cultures predict, we calculated $I_{\mathrm{mem}}$ and $I_{\mathrm{pred}}$ (Eq. 10) for each culture, for both stimulation modalities. The achieved predictive power ($I_{\mathrm{pred}}$) reached on average 66.7±8% of the theoretical maximum (R(D)), given what was memorized $I_{\mathrm{mem}}$ (Fig. 3G) when focally stimulated, and 30.4±6% when globally stimulated (Fig. 3H).

## Discussion

Several studies have addressed complex abilities and operations of the brain, like memory and prediction. It is generally presumed that these are capabilities of networks of neurons. We used in vitro networks of dissociated cortical neurons, which were shown to memorize external input, provided that the input generated new specific patterns that were not part of the spontaneous patterns before the stimulation (21, 22, 44, 23). Prediction, on the other hand, has mainly been studied theoretically (1–3), although some first experimental results have been published on retinal networks (5, 26–28). Prediction can be defined as the ability to reduce uncertainty on future external inputs, and is hypothesized to be a fundamental property of neuronal networks that constitutes our ability to successfully conclude everyday actions (1–3, 5). If this is true, other type of neural networks should also be able to predict. Our goal was to provide proof of principle that in vitro random neural networks are able to predict.

Here, we repeatedly stimulated in vitro networks of cortical neurons, and investigated whether recorded activity provided information about future stimulation. We additionally studied possible relationships between prediction and memory. Results show that cortical neurons can predict and that this ability is largely dependent on the network response to stimulation and short term memory.

Information about future focal, as well as global, stimuli were strongly determined by the distribution of interstimulus intervals (Fig. 2) and the associated intrinsic predictive information of the stimulus vector on itself (see Fig. S3 in Supplementary material). This, together with the dependency of MI on the presence of stimulus responses (Fig. 2), confirms that most information on the stimulus vector comes from stimulus responses, which should be clearly recognizable after digitization into 100 ms bins. Electrical stimulus pulses lasted less than 1 ms and were shown to directly activate only a small subset of all neurons (45), making it unlikely that these stimuli would be recognizable in the binarized activity signal without any further network responses. However, focal electrical stimulation typically induced network responses that lasted ≈150–250 ms (see Fig. S1 in Supplementary material) (46), with patterns that clearly deviated from spontaneous patterns, even after binarization. This induced network responses, plus immediate subsequent activity (up to ≈1 s after the stimulus) that can be seen as a form of short-term memory, given the timescale of seconds. Similar time scales of stimulus responses were observed following global optogenetic stimulation. Our results seem to indicate that given a stimulus response at time *t*, the network estimates that there cannot be other stimuli within the next 1,000 ms interval. This is visible in the almost flat MI curve for Δt≤1,000 ms (See Fig. 2). Moreover, the network predicts that the next stimulus most probably arrives after 1,100 ms, with decreasing probability for longer intervals (peak in ${\mathrm{MI}}_{\mathrm{future}}$ curves). Still, MI was always less than the entropy of the stimulus vector *S*. In silico simulations showed that networks predict optimally when ISIs were constant, with MI approaching the stimulus’ entropy (see Supplementary material, Fig. S4).

When a network receives stimulation, its response can be seen as the increased amount of action potentials recorded in the following 100–250 ms. The networks’ first step to memorize a stimulus is to be responsive. If the network responds then the given stimulus can disturb the balance in the network, leading to a change in functional connectivity (21). Fig. 2 shows that most of all information about future stimuli are contained in post-stimulus responses. Masked stimulus responses yielded a decrease in MI, with peak disappearing. Similar results were obtained when subtracting the intrinsic information in the stimulus itself. These results indicate that the network can predict if it is responsive to the stimulation. This shows that prediction depends on clearly recognizable stimulus responses, which, in case of electrical stimulation, probably depends on synaptic propagation. Electrical stimulation directly activates just a small subset of all neurons, and only during a minor fraction of the 100 ms analysis intervals. Blockade of action potential propagation in in vitro networks would lead to almost no activity, and even if direct activation of neurons might still occur, it would be difficult to discriminate between activity and stimulus artifacts. This hampers in vitro verification, but computational modeling confirmed that prediction depends on synaptic propagation (see Supplementary material, Fig. S4).

It has been hypothesized that the ability to make sensory predictions is related to short-term memory (3) but also long-term memory has been suggested to play a role in prediction (3, 47, 48). We found a clear correlation between prediction ($\Sigma {\mathrm{MI}}_{\mathrm{future}}$) and memory ($\Sigma {\mathrm{MI}}_{\mathrm{past}}$), indicating that networks need some memory of recent past stimuli to enable prediction of future stimuli. The amount of predictive information remained fairly constant during prolonged periods of stimulation. When stimulated focally, however, predictive information tended to become less dependent on short-term memory. Despite the decreasing importance of short-term memory, networks were still able to predict, suggesting that other mechanisms became dominant. This lower dependency on short-term memory is also reflected by the higher prediction efficiency upon focal electrical stimulation (Fig. 3G and H). Strikingly, in globally stimulated cultures, the dependency on short-term memory did not decrease with time, and, accordingly, prediction was less efficient in these experiments. Focal electrical stimulation induced long-term connectivity changes, but global optogenetic stimulation did not, or to a far lesser extent (Fig. 3B), which is in agreement with earlier findings (23). Repeated focal electrical stimulation induced long-term memory traces in cultured cortical networks within one or a few hours, and during the formation of memory traces the response patterns become part of the spontaneous patterns (21). This reduces the statistical temporal association between the stimulus response and future stimuli, and may explain the reduced dependency of prediction on the stimulus response pattern. However, the total predictive information did not decrease, and it remains unclear what aspect in recorded activity compensated for the decreased information in stimulus response patterns. Because long-term connectivity changes are required for long-term memory, it is probable that global optogenetic stimulation did not induce long-term memory traces. This suggests that the formation of long-term memory traces after several hours of stimulation may provide an additional factor to predict stimulation, that contributes to the higher efficacy of prediction during focal stimulation. These findings support the theory that sensory prediction is strictly related to the ability of the networks to memorize (3, 48). More detailed understanding of the underlying mechanisms will require further investigation, particularly the role of spike timing dependent plasticity (STDP), or at least the involvement of NMDA receptors seem plausible candidates for further research. Theoretical work showed that STDP is a fundamental mechanism in memory formation (49, 50).

We chose to vary the timing of stimulation, but not the location, whereas previous studies varied the location (5, 26–28). Retinal cells were shown to predict future positions of a moving bar, and it is possible that cortical networks would predict better if stimuli were spatially varied. Another factor to take in consideration is the slight decrease in responsiveness from the cultures beyond 12 h of stimulation (see Fig. S1 in the Supplementary material). Particularly when electrically (focally) stimulated, this decrease was around 50% in the last hours, which may have affected the relationship between prediction and short-term memory.

We conclude that the activity generated in random networks of cultured cortical neurons provides predictive information on future stimuli. This ability depends on short-term memory. When long-term memory traces are formed, prediction becomes more efficient and less dependent on short-term memory.

## Supplementary Material

pgad188_Supplementary_Data

## Data Availability

All experimental data are available on Dryad (https://doi.org/10.5061/dryad.18931zd2t).
