# Supplementary material for: Prediction in cultured cortical neural networks
Source: PNAS Nexus. 2023 Jun 27;2(6):pgad188. doi: 10.1093/pnasnexus/pgad188 (PMC10299080; doi:10.1093/pnasnexus/pgad188)
Supplement: pgad188_Supplementary_Data [file pgad188_supplementary_data.zip › PNASNEXUS-PNASNEXUS-2023-00060R-s01.pdf]

Supplementary Information for

Prediction in cultured cortical neural networks

Martina Lamberti, Shiven Tripathi, Michel J. A. M. van Putten, Sarah Marzen and Joost le Feber

Corresponding Author Joost le Feber.
E-mail: j.lefeber@utwente.nl

This PDF file includes:

Supplementary text
Figs. S1 to S5
Table S1
SI References

Supporting Information Text

Additional information on experiments acquisition

For our study we performed in total 19 experiments undergoing focal electrical stimulation. Among these experiments 11 had to be excluded for either lack of stimulus response in the last 2 to 3 h, or for technical issues related to the stimulator. For global optogenetic stimulation we used a total of 11 cultures. Also in this case 2 recordings were excluded for lack of response in the last hours. Table S1 gives an overview characteristics of the experiments included for analysis.

Additional analysis

Effectiveness of stimulation. Stimulus responses have been shown to decrease with high stimulation frequencies (1) To facilitate persistent responses, the average stimulation frequency was set to ≈ 0.2 Hz, with a minimum inter stimulus interval (ISI) of 1 s.To verify whether (focal electrical or global optogenetic) stimulation remained effective throughout the 20h stimulation periods, we summed all recorded activity in 5ms bins from 300ms before the stimulus until 500ms after to build post stimulus time histograms (PSTHs). Average PSTHs were computed per culture for each hour. We then calculated the area under the curve (AUC) in the interval between 15 ms and 500 ms after the stimulus to quantify the responsiveness to stimulation. The long duration of light pulses for optogenetic stimulation might impose a bias of direct activation, which could mask a possible decrease of network responses during the 20 h of experiments. We therefore added a curve of the time course of stimulus responses with AUC computed on the time interval $100 - 500$ ms after onset of the stimulus, thus excluding the period of direct activation. AUCs of all cultures were normalized to their value in the first hour, and then averaged across experiments. In addition, we analyzed whether response durations changed over time. The duration of stimulus responses was determined from PSTHs as the maximum time after the stimulus where the average PSTH was still higher than the averaged summed activity before the stimulation plus 5 times its standard deviation. Response durations were calculated per hour for each culture, and averaged across experiments.

Self prediction of stimulation signal. To test the hypothesis that the prediction mainly relies in the neuronal response to the given stimuli, we aim to calculate $MI_{\text{self}} = MI[S_{\text{t}}; S_{\text{t}+\Delta\text{t}}]$ and want to compare this to MI_{future}. Basically we applied MI between the stimulation vector shifted (backwards for prediction) and the same vector in its original time points (MI_{self}).

In parallel we also estimated MI from the probabilities of the different states present in the stimulation vector. This was possible because mutual information is related to the entropy, which is related to signal states probabilities.This is easily calculable if one knows the hidden Markov model generating the observed time series. The hidden Markov model that describes the data formally has an infinite number of states, though in our case, we truncate and approximate the model as having 1000 states. If we consider the stimulation vector (S) then there are two possible observations, $K_{\text{t}} = 1$ or $K_{\text{t}} = 0$. This means that there are four possible states with the following main probabilities: $P_{00}, P_{10}, P_{01}, P_{11}$. In our case the hidden Markov model, behind the the set of stimuli, can be described by the following labeled transition matrices:

$$T^{(0)}_{s,s'} = P(S_{t+1} = s, K_t = 0|S_t = s'). \quad [1]$$

$$T^{(1)}_{s,s'} = P(S_{t+1} = s, K_t = 1|S_t = s'). \quad [2]$$

Which added together bring to the total transition matrix

$$T_{s,s'} = T^{(0)}_{s,s'} + T^{(1)}_{s,s'} = P(S_{t+1} = s|S_t = s'). \quad [3]$$

The values that populate the labeled transition matrices are given in Ref. (2, 3). The interstimulus intervals come from a continuous-time fractal renewal process with $\phi(t) = \alpha(t/t_0)^{-(\alpha+1)}$ for $t \geq t_0$ with $t_0 = 1$ s, $\alpha = 1.05$, chosen because the excess entropy of this process is quite large and we are close to having a naturalistic autocorrelation function. This leads to (approximately) a discrete-time renewal process with $F(n) \approx \phi(n\Delta t)\Delta t$.

From this we can get the stationary distribution π normalising the eigenvector of eigenvalue 1 of T. Then the four main probabilities $(P_{00}, P_{10}, P_{01}, P_{11})$ of seeing one observation at time t and another at time $t + \Delta t$ are:

$$\begin{aligned} P_{00} &= P(K_t = 0, K_{t+\Delta t} = 0) = 1^T T^{(0)} T^{\Delta t - 1} T^{(0)} \pi \\ P_{01} &= P(K_t = 1, K_{t+\Delta t} = 0) = 1^T T^{(0)} T^{\Delta t - 1} T^{(1)} \pi \\ P_{10} &= P(K_t = 0, K_{t+\Delta t} = 1) = 1^T T^{(1)} T^{\Delta t - 1} T^{(0)} \pi \\ P_{11} &= P(K_t = 1, K_{t+\Delta t} = 1) = 1^T T^{(1)} T^{\Delta t - 1} T^{(1)} \pi. \end{aligned} \quad [4]$$

Then MI can be estimated based on these four main probabilities as follows:

$$
\begin{aligned}
&MI(K_t; K_{t+\Delta t}) = \\
&P(K_t = 0, K_{t+\Delta t} = 0) \log \frac{P(K_t = 0, K_{t+\Delta t} = 0)}{P(K_t = 0)P(K_{t+\Delta t} = 0)} \\
&+ P(K_t = 1, K_{t+\Delta t} = 0) \log \frac{P(K_t = 1, K_{t+\Delta t} = 0)}{P(K_t = 1)P(K_{t+\Delta t} = 0)} \\
&+ P(K_t = 0, K_{t+\Delta t} = 1) \log \frac{P(K_t = 0, K_{t+\Delta t} = 1)}{P(K_t = 0)P(K_{t+\Delta t} = 1)} \\
&+ P(K_t = 1, K_{t+\Delta t} = 1) \log \frac{P(K_t = 1, K_{t+\Delta t} = 1)}{P(K_t = 1)P(K_{t+\Delta t} = 1)}.
\end{aligned} \quad [5]
$$

Summation of these values yield to the amount of information that the stimulus provides on itself.

Assessing long term connectivity changes. For both focal electrical and global optogenetic stimulation experiments, we first applied conditional firing probability (CFP) method to estimates functional connectivity before (Baseline) and after (AftStim) the 20h stimulation period. With CFP models functional connectivity is estimated by calculating the probability that neuron j fires at t=τ ($0 \leq \tau < 500$ms), given that neuron i fired at t=0. Only active electrodes that recorded > 250 spikes in a period of 1 hour were considered to be active, and were used for the analysis (4, 5). To investigate possible changes in connectivity, both Baseline and AftStim recordings were divided into two blocks of 30 minutes. Euclidean distance was calculated between both connectivity matrices within Baseline. Following, Euclidean distances were calculated between both AftStim connectivity matrices and both Baseline matrices, and averaged. Only electrodes that were active during Baseline and AftStim were taken in consideration. We checked for possible differences between Euclidean distances within Baseline with the ones induced by the stimulation. One might argue that the presence of the AAV in cultures that were optogenetically (globally) stimulated and the absence in the other cultures could bias this comparison by introducing a larger spread in baseline. However, when both groups were analyzed separately, we still found significant connectivity changes only in electrically (focally) stimulated cultures.

How efficiently do cultures predict?. There is generally not enough capacity to store the entire past of the input, and despite this bottleneck, we still want to produce accurate information about the future of the input. To do so, we need to squeeze the past to predict the future, generally referred to as the predictive information bottleneck. The question is essentially, which aspects of the past do we need to keep in order to understand the future? Usually, more recent events are more important for prediction than earlier ones. It turns out, in a mathematically very precise way, that the necessary information to keep is information about the minimal sufficient statistics of prediction, the forward-time causal states S^+. Figuring out what aspects of these causal states are really necessary to keep, requires numerical computation. Let's let $\overleftarrow{S}$ be the past, and let's let $\overrightarrow{S}$ be the future of the input. Let $\widetilde{X}$ be the random variable for an hypothetical present neural activity. We would like to simply have:

$$I_{mem} = I[\overleftarrow{S};\widetilde{X}],\ I_{\text{pred}} = I[\overrightarrow{S};\widetilde{X}]. \quad [6]$$

The predictive information bottleneck simply says that we would like to minimize I_{mem} and maximize I_{pred} at the same time. If we set I_{mem} to 0, we have no information about the future, so I_{pred} will also be 0. In the other extreme, we might be able to capture all information about the future, so that I_{pred} is $I[\overrightarrow{S};\overleftarrow{S}]$.However, I_{mem} may be infinite then, because we might need to store the entire past. Hence, minimizing memory and maximizing predictive power implies a trade-off. The problem with the predictive information bottleneck as written, is that the entire past and entire future are impossible mathematical objects to work with. Recent work shows that we can replace the entire past of the stimulus by the time since last stimulus, S^+, and the entire future of the stimulus by the time to next stimulus, S^-, with minimum loss of information (example of S^+ and S^- in figure S5) (6).

$$I_{\text{mem}} = I[S^+;\widetilde{X}],\ I_{\text{pred}} = I[S^-;\widetilde{X}]. \quad [7]$$

Memory is correlated with resources used, time delays, and energy used, but not everything that is memorized contributes to prediction. Hence, memory represents costs, whereas prediction represents profit, but yet you need memory for prediction. Rate-distortion theory is a branch of information theory that connects minimum resources to maximum accuracy, as described in (7). To calculate this trade-off between memory and prediction, as in the predictive information bottleneck, we compute:

$$R(D) = \min_{p(\tilde{x}|s^+):I[\widetilde{X};S^-]\geq D} I[\widetilde{X};S^+], \quad [8]$$

where $\tilde{x}$ is a realization of $\widetilde{X}$, s^+ is a realization of S^+, and $p(\tilde{x}|s^+)$ is the conditional probability of neural activity given time since last stimulus. The way to read this equation is that out of all the conditional probabilities (neural activity patterns) that give a predictive power of at least D, we search for the one with minimal memory. D is the required predictive power, and $R(D)$ is the memory required to achieve that predictive power. The black dashed line (see Figure 3 G and H in the main text) is $R(D)$. According to the rate-distortion theorem, it separates achievable from unachievable combinations of prediction and memory. Thus, the black dashed line indicates optimum prediction, given certain amount of memory, and

can be obtained numerically using multivariable calculus techniques to turn the constrained optimization problem into an unconstrained optimization problem:

$$p_\beta(\tilde{x}|s^+) = \arg\min_{p(\tilde{x}|s^+)} I[\widetilde{X}; S^+] - \beta^{-1} I[\widetilde{X}; S^-] \qquad [9]$$

where $p_\beta(\tilde{x}|s^+)$ is the optimal neural activity pattern given past stimulation for a particular trade-off between memory and prediction, and β is a Lagrange multiplier that controls how highly we prize prediction compared to memory. In this equation, we are looking through all possible neural activity patterns and choosing the one that minimizes memory minus some constant (the Lagrange multiplier's inverse) times prediction. We then take partial derivatives and set them equal to 0, getting an equation that we then iterate (8):

$$p_\beta(\tilde{x}|s^+) = \frac{p_\beta(\tilde{x}) e^{-\beta D_{KL}[P(S^-|\widetilde{X}=\tilde{x})||P(S^-|S^+=s^+)]}}{Z_\beta(s^+)}. \qquad [10]$$

where D_{KL} is the Kullback-Leibler (7) and $Z_\beta(s^+)$ is a normalization factor. By iterating this for different β, and calculating $I[\widetilde{X}; S^+]$ and $I[\widetilde{X}; S^-]$ for that $p_\beta(\tilde{x}|s^+)$, you can trace out the gray line. The problem with this approach for our setup is that the time since last stimulus (S^+) and time to next stimulus (S^-) can be quite large. S^+ and S^- are continuous variables that can take any real value, which we translate here into discrete variables as we divide time in blocks of Δt, and count the blocks since/to last/next stimulus. In order to retain full predictive information in the large β limit, we need to take many time steps into account. Consequently, $P(S^-|\widetilde{X} = \tilde{x})$ and $P(S^-|S^+ = s^+)$ are very large matrices, leading to unfeasibly large matrix computation. To avoid this, we followed the approach by Ngampruetikorn and Schwab (9), which does not yield the entire curve, but a good estimate of the initial slope of the curve, using advanced perturbation theory techniques.Because the generalized Blahut-Arimoto algorithm can converge to local optima rather than the global optimum, There is anyway a possibility that the slope found by the generalized Blahut-Arimoto algorithm is not fully correct and would need random restarts to get correct.

Computational modeling. We used the *in-silico* model described by le Feber et al. (10) to assess the role of synaptic transmission in prediction. The model consists of 100 neurons, 80 were excitatory and 20 inhibitory. For comparison to *in vitro* data we selected 60 of these 100 neurons for further analysis. We used a set of neuronal parameters that follows the model by Izhikevic et al (11), which yields a mixture of all neuronal cell types present in the cortex, and gives simulations a good robustness against possible differences of cell properties. Connectivity was randomized, with an average connection density set to 50%. Synapses were plastic and expressed spike timing dependent plasticity (STDP) and short-term depression. Activity was initiated by synaptic noise, for details see (10).

We simulated 5 networks that received focal electrical stimulation. Stimulation was simulated by direct activation of a set 9 neurons that were randomly selected at the beginning of the simulation, and then kept constant during the entire simulation. We first validated that the model was able to reproduce prediction as found *in vitro*. Then, we simulated 5 regular networks that were stimulated at constant ISIs ($ISIfixed$, 1.1 s) to verify that that these are easier to predict. To determine the importance of synaptic propagation, we compared prediction in regular networks to that in 5 networks with all synaptic weights set to zero, stimulated with stochastic ISIs. For all simulated data we calculated MI_{past} and MI_{future} as described in the main text. To account for entropy differences between stimulation vectors, obtained results were normalized by the entropy of the given ISIs ($H_{ISI} = 0.15, H_{ISIfixed} = 0.44$).

Additional Results

Effectiveness of stimulation. During 20 hours of stimulation, the effectiveness of the applied stimulus might decrease, which would hamper interpretation of results. Before studying prediction and short term memory we verified that cultures remained responsive to the applied stimulation during the entire experiment. Area under the curve of stimulus responses remained fairly constant during the 20h of stimulation (focal electrical stimulation, $p > 0.9$, global optogenetic stimulation full PSTH, $p > 0.9$ and global stimulation after light off, $p > 0.9$), with a slight tendency to decrease towards the end (see Figure S1B). The duration of stimulus responses did not change during the 20 hours (focal electrical stimulation, $p > 0.8$, global optogenetic stimulation, $p > 0.7$). Stimulus responses always lasted less than 300 ms (Figure S1C and F). Responses to focal as well as global stimulation average did not significantly decrease throughout experiments, indicating that networks had sufficient time to recover between stimuli. In addition, network responses to both focal and global stimulation clearly outlasted the duration of the stimulus throughout experiments.

Intrinsic information of stimulation signal. The intrinsic predictive power of the stimulation signal itself follows the used distribution of inter-stimulus intervals. Figure (S3B) shows MI between the stimulus vector and time-shifted versions of itself (MI_{self}) calculated per each considered hour. It presents an obvious peak at $\Delta t = 0$ms, followed by a plateau between 0ms and 1000ms, and a second peak at 1100ms. Maximum MI, at $\Delta t = 0$ms, is around 0.15 bits which equals the entropy of the stimulus vector. The end of the initial plateau corresponds to the minimum ISI, and the second peak corresponds to the most probable inter-stimulus interval (Figure S3A). The same is seen when MI_{self} is calculated for the entire stimulation period (20h) (Figure S3C). On the other hand, Figure S3D shows the four main probabilities related to the possible states in the

stimulation vector (following Eq.4). The four probabilities taken into account ($P_{00}, P_{10}, P_{01}, P_{11}$) sum up to 1 within numerical errors. This led to the breakdown of MI into four partial MI contributors shown in Figure S3E. The final summation of the four MI contributors (Eq. 5) leads to the curve in figure S3F, which strongly resembles Figure (S3C).

Computational model. The computational model with regular synapses and stochastic ISIs was able to reproduce short-term memory (MI_{past}) and prediction (MI_{future}) as observed in *in-vitro* focally stimulated *in-vitro* networks (see Figure S4). Simulated networks without synaptic transmission showed strongly reduced short-term memory and prediction. When stimulated with constant ISIs the peaks of MI_{past} and MI_{future} increased, and both approached the optimum, given by the entropy of the stimulation vector (see Figure S4).

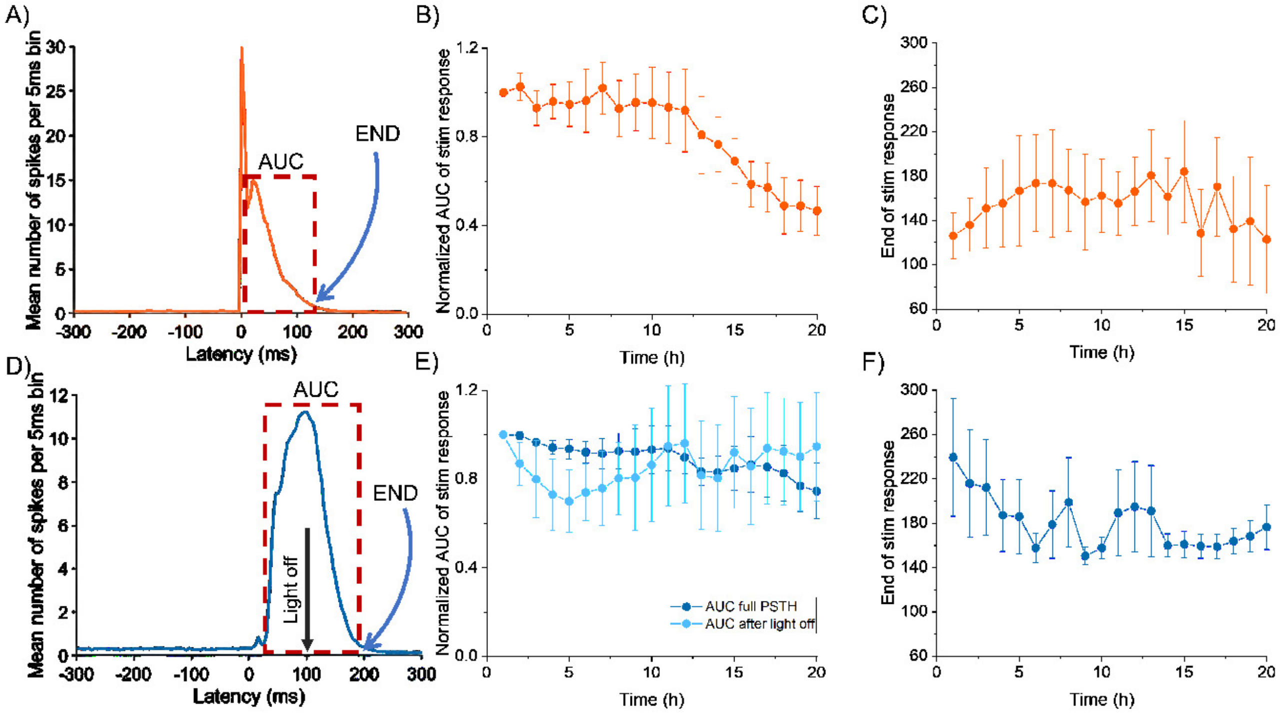

Fig. S1. Examples of post-stimulus responses and time course of effectiveness of stimulation. Top panels show effects of focal electrical stimulation (orange lines), bottom panels show effects of global optogenetic stimulation (blue lines). A,D) Example of post stimulus response. Red dashed rectangle indicates the interval for area under the curve (AUC) calculation. Blue arrow indicates point determined as the end of stimulus response. B,E) Mean AUC, averaged per hour. Before averaging across cultures AUCs were normalized to their value in the first hour of stimulation. In E) dark blue represents AUC in $15-500$ ms intervals, light blue represents AUC in $100-500$ ms intervals after onset of the light pulse. C,F) Mean duration of stimulus responses, calculated for each hour. Error bars show SEM and represent differences between cultures.

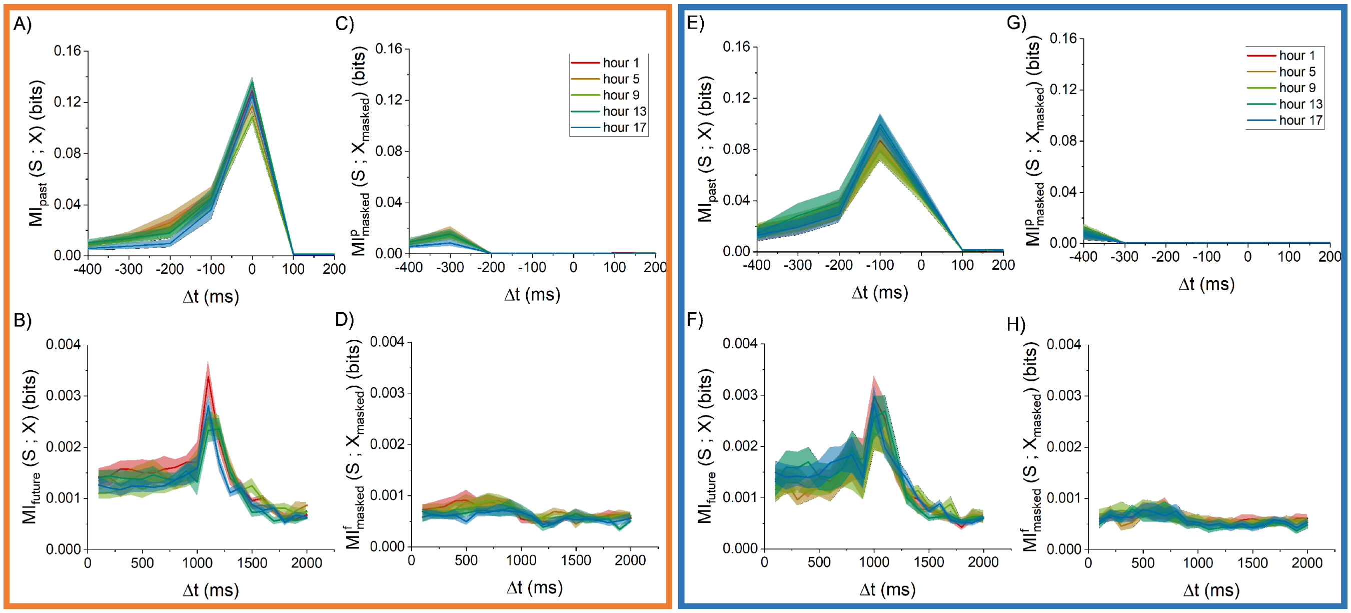

Fig. S2. Mutual information between activity and focal or global stimulation, shown per different hours. In the orange square (focal electrical stimulation results), left panels show estimates of short-term memory, MI_{past} (A), and estimates of prediction, MI_{future} (B), obtained from originally recorded data. In (A) the area between $\Delta t = 0$ ms and $\Delta t =$ -100 ms correspond to the immediate stimulus-response. While in (B) MI is maximum at $\Delta t = 1100$ ms. Panels (C) and (D) show MI^{p}_{masked} and MI^{f}_{masked} respectively. Here in both (C) and (D) the peaks at $\Delta t = 0$ ms and $\Delta t = 1100$ ms disappeared. Likewise in the blue square (global optogenetic stimulation results), left panels show MI_{past} (E) and MI_{future} (F) obtained from originally recorded data. In (E) the region between $\Delta t =$ -100 ms and $\Delta t =$ -200 ms correspond to the stimulus-response. While in (F) MI is maximum at $\Delta t = 1000$ ms. Panels (G) and (H) show MI^{p}_{masked} and MI^{f}_{masked}. Here in both (G) and (H) the peaks at $\Delta t =$ -100 ms and $\Delta t = 1000$ ms disappeared. In all panels different hours are represented by different colours. Shaded areas represent SEM.

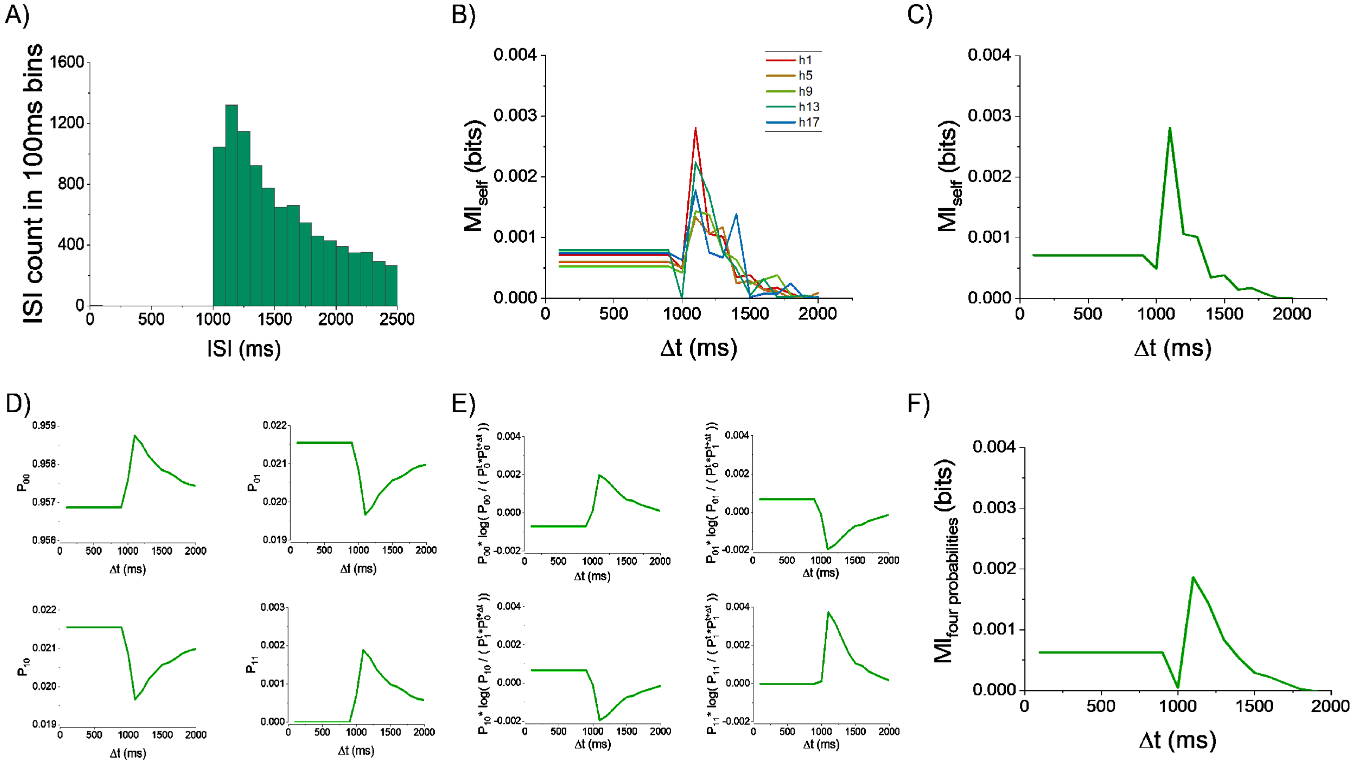

Fig. S3. ISI distribution and MI_{self} of the stimulation vector. A) shows the ISI distribution used in all experiments. B) shows MI_{self}, MI between the stimulation vector and time-shifted versions of itself, calculated for different hours. C) shows the total MI_{self} calculated using the entire stimulation period. The lower panels show the four probabilities (P_{00}, P_{10}, P_{01}, P_{11}) (D) and associated partial MI curves in panel E (see Eq.5). F) shows MI_{self} curve after summation of partial MI, as obtained from Eq. 5.

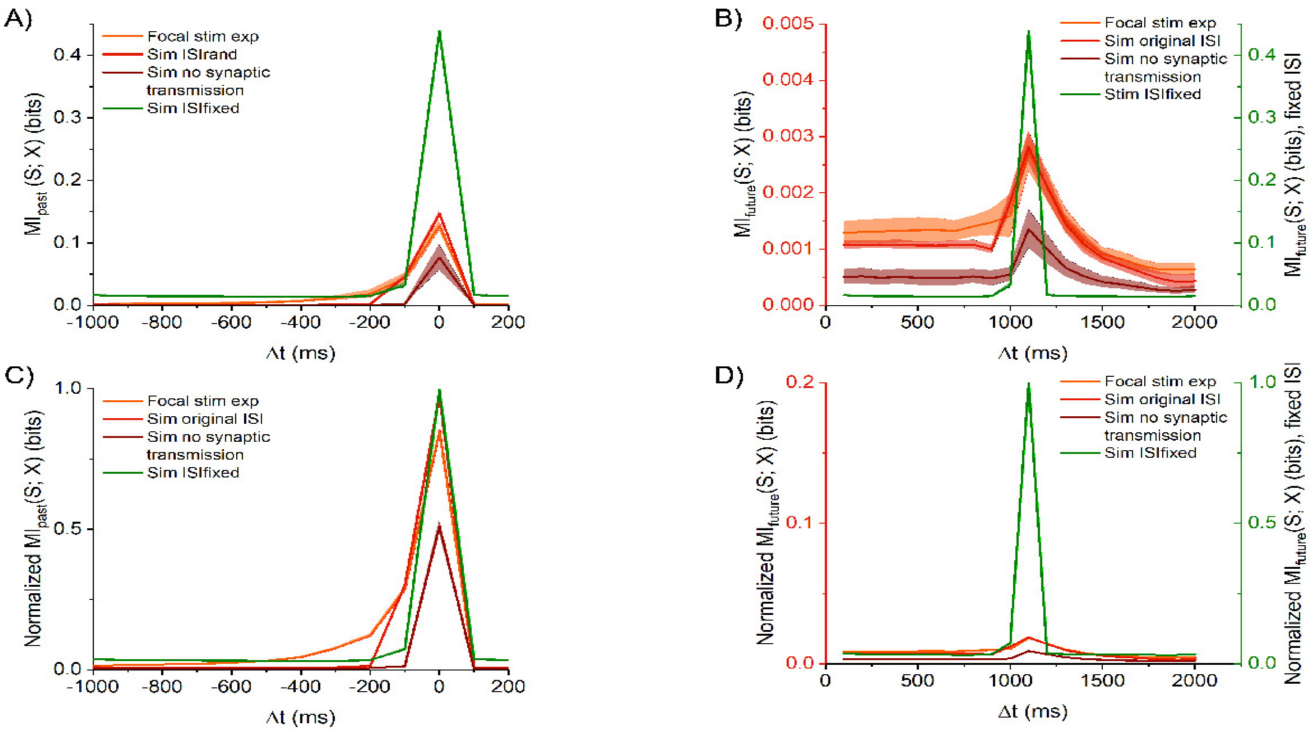

Fig. S4. Comparison between experimental data (focal stimulation) and in-silico simulations. Right panels show MI_{past} , left shows MI_{future} , in focally stimulated cultures (orange, n=8), simulated neural networks (light red, n=5), simulation with no synaptic transmission (dark red, n=5), simulated networks with $ISIfixed$ at 1.1 s (green, n=5). Top panels show mutual information in bits, bottom panels show mutual information normalized to the entropy of the stimulation vector S ($H_{ISI} = 0.15$, $H_{ISIfixed} = 0.44$). In B and D the left, red, vertical axes refers to the red and orange curves, the right, green, axes refers to the green curve.

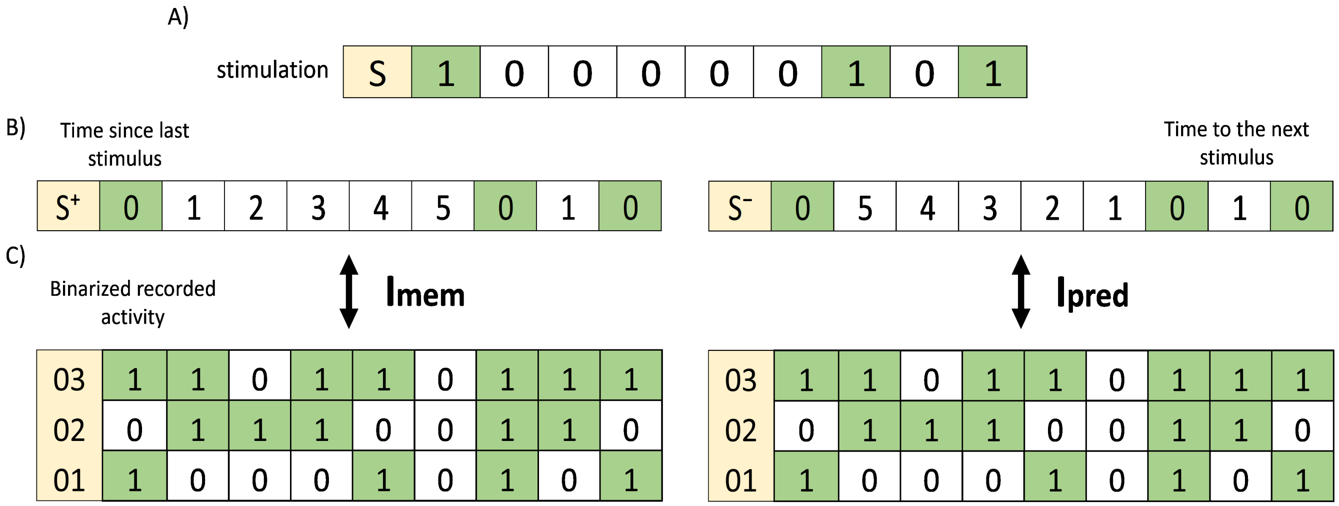

Fig. S5. Example to explain the calculation generation of S^+, S^- plus I_{pred} I_{mem} as defined in the main text. A) Is an example of a possible binarized stimulation vector S. Here a 1 indicates the presence of stimulus and zero otherwise. B) shows the transformation of S into S^+ (time since last stimulus) or S^- (time to the next stimulus). C) shows on the left calculation of I_{mem} applying MI between a possible binarized recorded activity and S^+. On the right calculation of I_{pred}.

	Electrical (focal) stimulation			Optogenetic (global) stimulation		
Experiments	active electrodes baseline	total spikes baseline	total spikes 1h after stim	active electrodes baseline	total spikes baseline	total spikes 1h after stim
1	38	254243	134044	39	600544	428000
2	27	100618	3348	29	201333	225702
3	26	243448	149324	53	511778	9406
4	52	252650	74424	31	242771	196204
5	48	393276	99332	22	50786	95051
6	33	76210	108268	28	127940	81889
7	32	76646	58794	51	391470	185844
8	41	157189	218444	25	149380	27648
9				46	492111	475087

Table S1. Experiments characteristics. For both type of stimulation (focal electrical stimulation and global optogenetic stimulation) the table shows the characteristics of the experiments included in the final analysis. the amount of active electrodes during the first hour of baseline recordings, and the amount of recorded spikes recorded 1h before and after the stimulation.

References

1. DA Wagenaar, R Madhavan, J Pine, SM Potter, Controlling bursting in cortical cultures with closed-loop multi-electrode stimulation. *J. Neurosci.* **25**, 680–688 (2005).
2. SE Marzen, JP Crutchfield, Informational and causal architecture of discrete-time renewal processes. *Entropy* **17**, 4891–4917 (2015).
3. SE Marzen, JP Crutchfield, Statistical signatures of structural organization: The case of long memory in renewal processes. *Phys. Lett. A* **380**, 1517–1525 (2016).
4. J le Feber, et al., Conditional firing probabilities in cultured neuronal networks: a stable underlying structure in widely varying spontaneous activity patterns. *J. neural engineering* **4**, 54 (2007).
5. M Lamberti, et al., Maximum entropy models provide functional connectivity estimates in neural networks. *Sci. Reports* **12**, 1–10 (2022).
6. SE Marzen, JP Crutchfield, Predictive rate-distortion for infinite-order markov processes. *J. Stat. Phys.* **163**, 1312–1338 (2016).
7. M Thomas, AT Joy, *Elements of information theory.* (Wiley-Interscience), (2006).
8. N Tishby, FC Pereira, W Bialek, The information bottleneck method. *arXiv preprint physics/0004057* (2000).
9. V Ngampruetikorn, DJ Schwab, Perturbation theory for the information bottleneck. *Adv. Neural Inf. Process. Syst.* **34**, 21008–21018 (2021).
10. J le Feber, T Witteveen, TM van Veenendaal, J Dijkstra, Repeated stimulation of cultured networks of rat cortical neurons induces parallel memory traces. *Learn. & memory* **22**, 594–603 (2015).
11. EM Izhikevich, Simple model of spiking neurons. *IEEE Transactions on neural networks* **14**, 1569–1572 (2003).
